# The chromosomal origin of replication as the basis for the spatio-temporal biology of bacteria

**DOI:** 10.3389/fmicb.2026.1786250

**Published:** 2026-04-14

**Authors:** Vic Norris, John Herrick, Masamichi Kohiyama

**Affiliations:** 1 Bacterial Communication and Anti-infectious Strategies (UR4312), University of Rouen Normandie, Evreux, France; 2 Institut des Systèmes Complexes Paris Île-de-France, Paris, France; 3 Independent Researcher, Paris, France; 4 Universite Paris Cite, CNRS, Institut Jacques Monod, Paris, France; 5 Association pour Developpement Culturel et Scientifique d'Abondance (ADCSA), Abondance, France

**Keywords:** bacterial cell cycle, condensate, DNA replication, hyperstructure, initiation, origin, paradigm, phase transition

## Abstract

Spatial biology depends on the structural dynamics of the constituents of the cell. We propose that such dynamics during the cell cycle depend to a large extent on the origin of replication and its role in the trajectory of a replication hyperstructure. To explore this *ori-Centric* view, we adopt both historical and speculative approaches to the initiation of chromosome replication, focusing on *Escherichia coli*. We relate these approaches to the framework of concepts, results, and procedures within which science is structured; this framework can be considered a paradigm or a “thought style” that sometimes undergoes a major shift. We suggest that a paradigm shift is underway in the study of the cell cycle as shown by the increasing contribution of physical chemistry and, in particular, phase transitions.

## 1 Introduction

The passage through the cell cycle exerts a major influence on the spatio-temporal structure of the bacterial cell—and vice versa. The chromosome, which when associated with the coupled processes of transcription and translation, can take up half or more of the volume (Woldringh, 2024). In *E. coli*, this chromosome is duplicated and segregated by processes that involve protein clusters (Hiraga et al., 1991; Nolivos et al., 2016) and membrane attachments via transertion (Binenbaum et al., 1999; Gorle et al., 2017; Spahn et al., 2025). Finally, the act of cell division also involves a major remodeling of the envelope and cytoplasm.

The first event in the cell cycle is generally considered to be the initiation of chromosome replication. Our now detailed understanding of this event has been achieved mainly thanks to hypotheses and techniques derived from molecular biology and biochemistry. Two of the most powerful of these hypotheses include the Replicon Theory (Jacob et al., 1963) and the “Initiation Mass” (Donachie, 1968): the former has successfully guided isolation of the molecular elements of replication initiation, whereas the latter has contributed to the determination of the timing of their action in the cell cycle (see below). In parallel, our understanding of the cell cycle has been increasingly influenced by concepts and techniques derived from physics and physical chemistry such as viscoelasticity

(Ferry, 1980), exclusion volumes (de Gennes, 1979), phase transitions or shifts (Stanley, 1971), entropic polymer demixing (Flory, 1942), Langmuir films (Möhwald, 1990), Turing structures (Turing, 1952), microfluidics (Whitesides, 2006), etc.

**Figure 1:**
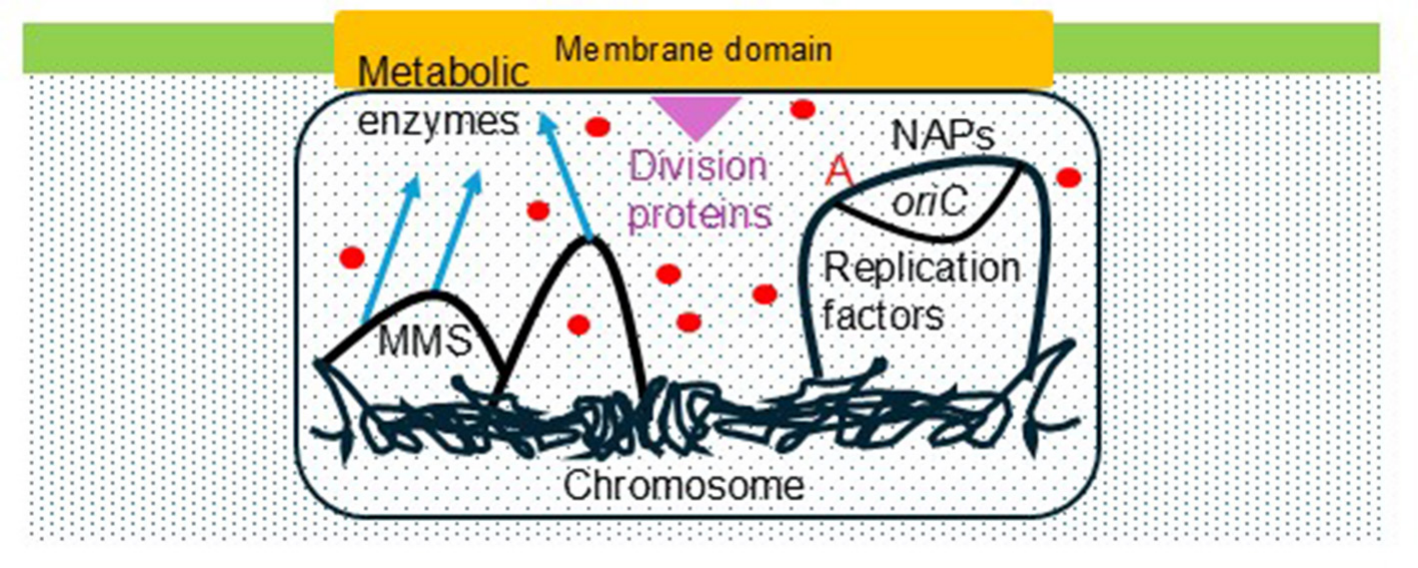
Composition of a speculative, initiation hyperstructure. DnaA (red A) is shown binding to the origin of replication (*oriC*). Replication factors include proteins like DnaB, DnaC, DnaG and ribonucleoside diphosphate reductase. NAPs include IHF and FIS. Division proteins include MinC and FtsZ (purple triangle). Metabolic enzymes include some of the enzymes of the Central Carbon Metabolism and the periplasmic AphA. Coupled transcription-translation (blue arrows) links genes to the hyperstructure; these genes include those in the Macromolecular synthesis operon (MMS) like DnaG. Small molecules and ions such as cAMP and Ca^2+^ are shown as red circles. A membrane domain with a particular phospholipid composition (e.g., enriched in cardiolipin) is an integral part of the hyperstructure. The hyperstructure has a lower proportion of free water than its surrounding environment (small dots).

Here, we argue that a paradigm shift is underway in the field of the bacterial cell cycle. This new paradigm is taking the form of a cell cycle dominated by the dynamics of big structures of molecules and macromolecules termed hyperstructures that (1) can have complex, changing structures and compositions, (2) may have their own physical (e.g., vibrational) or chemical (e.g., based on a small molecule) “language,” and (3) owe their existence in part to phase transitions/shifts, which are part of the armory of physical chemistry (Norris et al., 1999, 2007b,a). One of the advantages of the hyperstructure concept is that it can bring together a wide variety of different results. Hyperstructures include the putative initiation hyperstructure, which comprises replication enzymes, metabolic enzymes, various genes and membrane (Figure 1). Here, we focus on the concept of an initiation hyperstructure, firstly relating it to the history of the origin of replication of *Escherichia coli*, secondly, showing how it could integrate a wide variety of apparently unconnected results, and, thirdly, speculating on its future development (Norris et al., 2002; Norris, 2011).

## 2 Historical overview of the isolation of *oriC*

Relatively few modern biologists realize that the concept of the origin of replication was first proposed in the Replicon Theory of (Jacob et al. 1963). In this hypothesis, they proposed that the replication of the chromosome starts from a single origin after a specific interaction between this origin and the “initiator protein.” Importantly, they proposed that this origin-initiator interaction is essential for the maintenance of the chromosome in the bacterial cell. Moreover, the position of the origin corresponded to a specific genetic locus non-randomly located on the chromosome. They formulated this hypothesis during a discussion on the sexual conjugation of *E. coli* between an *Hfr* strain (which contains a conjugative plasmid like the F plasmid integrated in the chromosome) and an *F-* strain (which does not contain the F plasmid).

Soon after the publication of the Replicon Theory, thermosensitive mutants were obtained in *E. coli* that were consistent with the prediction of the existence of the initiator protein as they had the phenotypes expected for a defect in the initiation of chromosome replication (Kohiyama et al., 1963). The location of the mutations was determined and the gene to which they mapped was named DnaA Hirota et al., (1970). Classical enzymological studies on the isolation of the DnaA protein proved relatively unfruitful. In contrast, considerable advances were made on the genetic aspects of the initiator and on the origin of replication. In 1963, Sueoka's group measured gene dosage by using DNA transformation, which enabled them to reveal a sequential duplication of the genes in a culture of germinating spores of *Bacillus subtilis* (Yoshikawa and Sueoka, 1963). Using an *E. coli* strain that had integrated phage Mu at different loci, Caro's group used DNA-DNA hybridization and cultures synchronized by amino acid starvation to determine DNA replication as starting at *ilv* 74 min (Bird et al., 1972).

Different copies of the *F* plasmid compete for the same duplication machinery and hence cannot be readily maintained in the same cell, that is, they are incompatible. Hiraga obtained an *F* plasmid construct containing the origin region and found it could be maintained in an *Hfr* strain (such strains contain an *F* plasmid integrated into the chromosome), which meant that Hiraga's *F* construct was using an origin that differed from the *F* origin of replication; this was in accordance with the Replicon Theory and he named this origin, *oriC* (Hiraga, 1976).

With the advent of genetic engineering, Worcel's group synchronized replication via temperature shifts in a *dnaC* culture and, following radioactive pulse-labeling, isolated the restriction enzyme fragments containing the highest levels of radioactivity, which allowed them to attribute the replication initiated at *oriC* to a 1.3 kb fragment (Marsh and Worcel, 1977).

A relatively precise construction of an *oriC* plasmid based on the Replicon Theory was first achieved by Hirota's group by taking the *oriC* region (as defined by Worcel's group) to allow replication and combining it with a gene encoding ampicillin resistance to allow selection of the resultant plasmid (Yasuda and Hirota, 1977). Independently of the above, von Meyenburg in Maaloe's laboratory was working with transducting bacteriophages to study a region containing ribosomal genes when he found a segment that, when incorporated into a lambda phage, allowed its maintenance in a lysogenic strain that would normally be immune to another copy (von Meyenburg et al., 1978).

The widespread adoption at this time of the Maxam-Gilbert method of DNA sequencing had the consequence that the definition of *oriC* by classical genetical mappings was no longer considered sufficient for publication. Instead, base-sequence analysis was required. In Japan, there was only one laboratory (headed by Takanami at Kyoto University) that could sequence DNA. Hirota, having contacted this laboratory first, succeeded in getting the sequence (Sugimoto et al., 1979) whilst Hiraga, despite his advance, was unable to get it because his contacting Takanami came too late. In Europe, von Meyenburg, facing the same kind of problem, asked Messer (Max Planck Institute, Berlin) to collaborate with him scientifically and financially on an *oriC* sequencing project (von Meyenburg et al., 1978, 1979; Meijer et al., 1979). At the Cold Spring Harbor Symposium in 1978 (Witkowski, 1978), these groups presented the base sequences of *oriC* and noted the “high degree of repetitiveness due to both inverted and direct repeats” that, potentially, could lead to secondary structures such as loops, hairpins, cruciform structures etc. (Meijer et al., 1979), though the roles of these sequences in the initiation of replication were not understood at the time.

**Figure 2:**
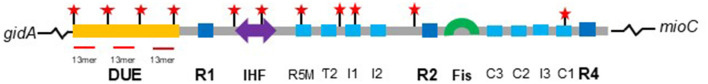
Location of different elements in *oriC*. The high affinity binding sites (dark blue DnaA boxes, R1, R2, and R4) are in bigger letters than the DnaA boxes with lower affinity (light blue boxes). Dam-binding sites are indicated by red stars (adapted from Hansen and Atlung, 2018). DUE, DNA Unwinding Element containing AT-rich 13mers; Fis, Factor for inversion stimulation (green arc); IHF, Integration Host Factor binding site (purple double-headed arrow; Kasho et al., 2023).

Kornberg's group made a breakthrough by creating a system for the replication *in vitro* of an *oriC* plasmid using a cellular fraction of a *polA* mutant (Fuller et al., 1981). The same sort of extract obtained from a *dnaA polA* mutant was inactive, which allowed them to perform complementation experiments using fractions of an extract of a wildtype cell. This enabled them to isolate the DnaA protein. As predicted for the initiator protein in the Replicon Theory, they found using DNase protection experiments that DnaA does indeed bind *oriC*; binding occurred at mainly four sites which they termed “DnaA boxes” and which had the consensus sequence TTATXCACA (Fuller and Kornberg, 1983). Finally, after performing deletion experiments using an *oriC* plasmid, Hirota's group defined the *oriC* sequence as a segment of 245 bp (Tabata et al., 1983; Figure 2).

## 3 *oriC* as an element for both positive and negative control of replication

Meselson and Stahl (1958) investigated the replication of DNA and found it to be semi-conservative whilst Cairns obtained an EM picture of one replicating “ring” of the chromosome of *E. coli* (Cairns, 1963) that he then posted to Jacob, who appreciated it very much as supporting evidence for the Replicon Theory. These results indicated that, in slow growth conditions, the initiation of replication occurs just once in a cell cycle after a long period in which a second initiation does not occur. *oriC* is the key element in this regulation (see below).

### 3.1 Positive control

The nucleotide sequence analysis of o*riC* revealed the presence of a “Duplex Unwinding Element” (DUE) containing three sets of AT-rich 13mers (which are characterized by a low melting temperature, a propensity to breathe, a high torsional pliability and a reduced stacking energy) in the left extremity of *oriC*, followed by a DnaAR1 box, an IHF (Host Integration Factor) binding site, a FIS (Factor for Inversion Stimulation) binding site, and high affinity (R1, R2, and R4) and low affinity (r2, l1, l2, c3, c2, l3, and c1) DnaA binding sites; ATP induces the formation of oligomers of DnaA (Felczak and Kaguni, 2004), which recognize the low affinity sites (Miller et al., 2009; Figure 1).

The opening of *oriC* is required for the loading of the DnaB helicase. Thus, DnaA plays an analogous role to the multi-component Origin Recognition Complex in eukaryotes: loading essential helicases to actually initiate DNA synthesis. Katayama's group analyzed o*riC* opening (Sakiyama et al., 2022). The first step is DnaA polymerization (into a spiral filament) between the strong R1 and R4 binding sites due to oligomerisation of the protein with the aid of ATP. IHF binds its site to bend *oriC* by 180°, resulting in DnaA binding (via Val121 and Arg245) to the T-rich strand of the opened DUE (Ozaki et al., 2008). Katayama and collaborators proposed a scheme for ssDUE recruitment by DnaA involving an IHF-bend inducing DUE opening (Ozaki and Katayama, 2012; Sakiyama et al., 2017) and this was subsequently termed the “Loop-back model” and adapted for R1 plasmid replication as explained in detail by Wegrzyn et al. (2023). Grimwade and Leonard (2021) also presented a model showing how FIS participates in the regulation of initiation.

### 3.2 Negative control

The term “eclipse” describes the minimum time permitted between successive replications (Nordstrom, 1983; von Freiesleben et al., 2000; Zaritsky et al., 2017). In *E. coli* [but not in *B. subtilis* (Jameson and Wilkinson, 2017)] this involves the formation of hemimethylated DNA. Russell and Zinder showed that hemimethylation prevents DNA replication in *E. coli* (Russell and Zinder, 1987). The DNA adenine methyltransferase methylates DAM sites, which are GATC sequences, and one of the intriguing features of the nucleotide sequence of *oriC* is the high frequency in it of the DAM sites (Sugimoto et al., 1979) and, subsequently, the Messer et al. (1985) group showed the inability of a *dam* mutant to maintain *oriC* minichromosomes. Boye's group found that these sites played an important role in initiation since, in a *dam* mutant, the timing of initiation was perturbed whilst cells having two sets of chromosomes failed to initiate replication of them simultaneously (Boye et al., 1988).

Schaechter's group tested the Replicon Theory's proposal of the importance of the cell membrane in cell cycle control; they examined interactions between the outer membrane and *oriC* and found a tight binding of *oriC* to the membrane only when *oriC* was in a hemimethylated state for only 1/3 of the cell cycle (Ogden et al., 1988). Then Landoulsi et al. (1990) demonstrated that this membrane preparation inhibits *in vitro* replication of *oriC* plasmid only in its hemimethylated form. Thus, the combination of the membrane and hemimethylated *oriC* constitutes a negative control mechanism of initiation of replication in *E. coli* that helps with the segregation of the chromosome.

The major element of this negative control system is the SeqA protein (Slater et al., 1995; Brendler et al., 1995), which binds to hemimethylated DAM sites with a higher affinity than to the unmethylated or the fully methylated DAM sites. Other factors are involved in sequestration, since a delta *seqA* strain retains a capacity, albeit diminished, to sequester *oriC* (Lu et al., 1994). This negative control system is considered to comprise a sequestration mechanism that prevents premature reinitiations at *oriC* and organizes the orderly segregation of newly synthesized DNA (Sawitzke and Austin, 2001; Waldminghaus et al., 2012). The nature of the membrane responsible for this sequestration was investigated by d'Alencon et al. (1999) who found, using a *dnaC* mutant synchronized for replication by a temperature shift, that *oriC* sequestration by the outer membrane varied during cell cycle: this sequestration activity was greatest for the membrane extracted during the sequestration period and gradually decreased thereafter. Moreover, this variation ran parallel to the presence of SeqA in the membrane (d'Alencon et al., 1999), consistent with the membrane again playing an important role in sequestration. Another explanation for how SeqA exerts a negative control is by preventing the DUE from opening. This is based on the fact that the DUE is a region highly protected by SeqA from DNase digestion (Taghbalout et al., 2000).

Leonard's group investigated the mechanism responsible for this negative control over initiation by performing *in situ* DMS footprinting (DMS methylates the G in the DnaA box when it is covered by DnaA): they found using a temperature-shifted *dnaC* that only the R1, R2, and R4 boxes were occupied during the sequestration period but not the R5M, l2, and l3 sites (Nievera et al., 2006).

### 3.3 The timing of initiation

What are the mechanisms by which the initiation of replication occurs at a defined moment of cell cycle? One classical parameter is the “Initiation Mass” proposed by Donachie (1968) according to which initiation occurs when a bacterium attains a particular ratio of mass to the number of chromosomal origins of replication that is independent of its growth rate. In consequence, slowing the replication fork rate in the mother cell results in it having a larger mass at the time of initiation (Zaritsky et al., 2006). Since the mass of the cell is closely linked to protein synthesis, addition of the protein synthesis inhibitor, chloramphenicol, inhibits initiation of replication (Maaloe and Hanawalt, 1961; Lark and Renger, 1969). The mechanism underlying the Initiation Mass was proposed at the time to be either the accumulation of an activator of initiation (Donachie, 1968) or the dilution of a repressor of initiation (which had worked well for the initiation of plasmid replication; Pritchard et al., 1969). DnaA became the leading candidate for the role of initiator protein as presented in the “Titration model” (Hansen et al., 1991). To investigate whether DnaA is the limiting factor for initiation in cells in which RNA or protein synthesis is inhibited, *dnaA* was expressed using a T7 RNA polymerase that is resistant to rifampicin (which inhibits *E. coli* RNA polymerase); this showed that expression of *dnaA* could relieve initiation inhibition (to some extent, even in the presence of an inhibitor of protein synthesis; Riber and Lobner-Olesen, 2020).

This result would be consistent with the importance of the amount of DnaA available for initiation as proposed in the “Titration model” (Hansen et al., 1991) providing it is assumed that the chance of initiation occurring is at a maximum when *dnaA* transcription is maximum. However, single cell analyses based on TRIP (Transcription-Replication Interaction Profile) led to the conclusion that, contrary to the gene dosage effect observed in most *E. coli* genes, *dnaA* is expressed maximally *after* initiation of replication in the second half of the cell cycle, when SeqA-free *oriC* is expected to rebind DnaA and license the next round of initiation/replication before cell division (Pountain et al., 2024).

The Initiation Mass was also studied at the single cell level using microfluidics, which showed that initiation occurred after an increase in cell length that had a constant value and that was independent of cell size at birth or the growth rate (Witz et al., 2019); moreover, *oriC* stayed around the same cellular spot during half the period of the cell cycle (as revealed by FROS: the insertion of a fluorescent protein binding site close to *oriC*) and this spot corresponded roughly to the site of the FtsZ constriction ring.

Simple models in which initiation results from the accumulation of an initiator or the dilution of a repressor with respect to the number of origins predict that cloning *oriC* into a multicopy plasmid (to make a minichromosome) should either postpone initiation or cause excess initiation. Disconcertingly, it turned out that cells could harbor scores of minichromosomes without perturbing the initiation of the chromosome, suggesting that it is not the mass per *oriC* that determines the Initiation Mass. Worse, the replication of these minichromosomes themselves was not only initiated synchronously but initiated at the same time as that of the chromosome itself (Leonard and Helmstetter, 1986; Koppes and von Meyenburg, 1987); note too the later findings that the *E. coli* chromosome can still be replicated with two origins functioning at the same time, particularly if (1) replication-transcription collisions are minimized, (2) the trapping of replisomes in the terminus is avoided, and (3) dNTP levels are raised (Ivanova et al., 2015; Dimude et al., 2018; Skovgaard, 2025).

Hypotheses mutate to accommodate data that risks falsifying them (Norris, 2024a). In the case of DnaA as the initiator protein, the solution was to have DnaA binding firstly to high affinity binding sites throughout the chromosome and only then binding to low affinity sites in *oriC* that initiated replication (Hansen et al., 1991); thus the replication of the chromosome itself became a central element in timing. Other hypotheses also invoke the chromosome as integral to cell cycle timing as in the case of transcriptional sensing where an increasing density of RNA polymerases transcribing a “sensor” gene triggers replication (Norris, 1995). Such sensing is consistent with modeling (Lin and Amir, 2018) and with recent results on genome dilution (Makela et al., 2024); it might even help explain replication from the *oriK*s whereby the RNA-DNA hybrids or R-loops that can result from transcription serve as the basis for initiation (Ogawa et al., 1984; Maduike et al., 2014) rather than *oriC* (Kouzminova et al., 2025). Unfortunately, all these chromosome-based ideas fall foul of the key experiment of Eliasson and Nordstrom (1997). Their experiment was based on integrative suppression in which a plasmid integrated into the chromosome can suppress an initiation defect (Nishimura et al., 1971); they found that in an integratively suppressed *recA* strain of *E. coli*, in which replication occurred at random, minichromosomes were replicated together as if the initiation signal were being given normally, again suggesting that the normal initiation from *oriC* is more a consequence of some factor with which the Initiation Mass is correlated that does not involve the replication of the chromosome itself from *oriC* (Eliasson and Nordstrom, 1997).

### 3.4 Cellular location of *oriC*

Since newly replicated *oriC* interacts with an outer membrane fraction (see Section 3.2), *oriC* might be restricted to one position in the cell without being able to move away, at least, during the sequestration period. TopoIV is responsible for removing the precatenates that result from the newly replicated chromosomes being intertwined, which is needed to allow them to be segregated [Cebrian et al., 2015; for other references see (Helgesen et al., 2021)]. One possibility is that sequestered *oriC* is bound by proteins that inhibit TopoIV activity like perhaps SeqA, which inhibits TopoIV binding *in vitro* (Kang et al., 2003) and which, when overproduced, increases the cohesion period (Joshi et al., 2013). Sherratt's group determined the cellular location of *oriC* as well as its movement during cell cycle in cells embedded in soft agar as revealed by a fluorescent construct (TetR-mCerulean attached to *tetO*) inserted 15 kb from *oriC* (Reyes-Lamothe et al., 2008); they found that, in a *dnaC* culture synchronized at the initiation stage, cells had one focus with replisomes and that quickly (at around 1/5 of the cell cycle) this focus split into two foci moving rapidly into opposite directions. Such *oriC*-replisome splitting had already been reported a few years before by Kleckner's group using fixed cells (Bates and Kleckner, 2005). Subsequently, Skarstad's group localized a SeqA fluorescent focus with *oriC* to midcell before it split into two foci that moved quickly to $\frac{1}{4}$ and $\frac{3}{4}$ positions at the end of the sequestration period (Fossum-Raunehaug et al., 2014).

The results of the above experiments can be revisited in the light of more recent experimentation using microfluidics. Witz et al. (2019) found that *oriC* is located at midcell during half the cell cycle and, significantly, that this position corresponded approximately to the site of FtsZ constriction. In determining the cellular location of both *oriC* (via FROS) and the replisome (via DnaN-mCherry), Elf's group found that a small cell (of around 2 μm) had two spots corresponding to *oriC* with replisome at the $\frac{1}{4}$ and $\frac{3}{4}$ positions (Gras et al., 2024). The fairly stable positions of these spots are difficult to attribute to an association with membrane as they appeared to lie on the long axis of the cell as found previously by Sherratt's group (Reyes-Lamothe et al., 2008).

### 3.5 A hyperstructure for initiation control?

From the observations described in the preceding sections, it seems reasonable to suppose that in slowly growing cells in the buildup to replication, *oriC*, sitting around midcell, is opened mainly by DnaA action, and is then replicated to yield the hemimethylated form that is sequestered principally by SeqA present in a membrane complex for one third of generation time. Then the *oriC* complex splits into two complexes that move relatively quickly, one to the $\frac{1}{4}$ position and the other to the $\frac{3}{4}$ position, separating from SeqA in the process, which stays in the central position. The *oriC* sequestration site is marked by the FtsZ constriction ring for cell division. Significantly, physical chemical changes in the membrane revealed by the lipophilic styryl dye FM 4–64, believed to correspond to the formation of membrane domains, are apparent very close in time and position to the start of separation of the nascent daughter chromosomes and these positions correspond to the future division sites (Fishov and Woldringh, 1999). All these complex processes and events have to be orchestrated—how might this be done? The concept of hyperstructure may prove helpful here.

Back in 1961, Mitchel proposed the notion of a hyperstructure connected with the membrane in order to explain his chemiosmotic theory (Mitchell, 1961). Over 40 years later, hyperstructures were again proposed as spatially extended assemblies of molecules and macromolecules responsible for performing many functions (Norris et al., 2007a). They include the condensates produced by phase separation (a phase separation is a phase transition in which occurs a transition from a single liquid phase of mutually soluble components to two or more distinct liquid phases). For example, the condensates that result from phase separation in bacteria are proposed to include the single-stranded DNA binding protein, SSB (Harami et al., 2020; Ecsedi et al., 2025), Noc (Babl et al., 2022), FtsZ (Monterroso et al., 2019), and Dps and HU (Gupta et al., 2023), among others. At a higher level than that of hyperstructures, it should be noted that the nucleoid itself is phase-separated from the surrounding “riboid” or non-DNA phase of the cytoplasm where most translation and metabolism occur (Kuzminov, 2024); it could be argued that this high level phase separation, which gives rise to the nucleoid periphery, is both created by hyperstructures and helps create hyperstructures by acting as a surface facilitating their assembly, as may be the case for transcription factories and the SSB- and SeqA-dependent replication factories.

Intrinsically disordered proteins (IDPs) and proteins with intrinsically disordered regions make up half the human proteome and constitute the “unfoldome” (Uversky, 2010). It has been proposed that an “epigenetic dimension of protein structure” exists in which “water, membrane lipids and ligands are… parameters that can control the structure of a protein (or a protein domain) in a particular spatiotemporal environment” (Fantini et al., 2025). IDPs can play a major role in phase transitions, particularly when the availability of water is low (Elder et al., 2025). Significantly, SSB is an IDP that forms a condensate (Kozlov and Lohman, 2024) whilst DnaA also contains an intrinsically disordered region (Hou et al., 2022). It seems therefore plausible that phase transitions have major effects on the assembly and disassembly of hyperstructures; this can be taken further with the idea that cells actually exploit phase transitions in various ways, one of which is to govern the cell cycle (Norris, 2024b).

In the hyperstructure approach to the cell cycle, the initiation of chromosome replication depends on the assembly and functioning of a hyperstructure that comprises DnaA, SeqA, the classical replication enzymes (like DnaBC, DnaG, and DnaE), FtsZ, nucleoid-associated proteins like HU and IHF, SSB, and glycolytic and other enzymes connected to the membrane (Herrick et al., 2025). It is conceivable it contains the genes that encode such proteins as DnaG and the glycolytic proteins that interact with them like pyruvate kinase in *B. subtilis* (Soultanas and Janniere, 2023); given the many interactions between the enzymes responsible for ribosomal, replication and other functions in *E. coli* (Morcinek-Orlowska et al., 2023), it is even possible that the initiation hyperstructure contains ribosomal genes and proteins or that it has direct interactions with a ribosomal hyperstructure (Herrick et al., 2025). These speculative possibilities might help to explain the interaction between DnaG and PykA as needed for the colocation within a common hyperstructure of pyruvate kinase as a GTP generator (She et al., 2025) and the GTPase FtsZ as a GTP consumer (de Boer et al., 1992). They might also help to explain the involvement of the outer membrane in the initiation of replication given that the chromosomal position of *pykF*, which encodes pyruvate kinase I, is next to that of the *lpp* gene, which encodes the abundant Braun's lipoprotein: the location and degree of expression of *pykA* might therefore be coupled with that of *lpp* and connected to the envelope via transertion as part of the initiation hyperstructure. It should be noted that there is evidence for the existence of a hyperstructure for the synthesis of the outer membrane proteins with many possible interactions between its constituents including, perhaps, between proteins involved in division, FtsQ and DamX, and those involved in outer membrane synthesis, SurA and BamA, respectively (Gao et al., 2022).

In this hyperstructure hypothesis, the dynamics of the initiation hyperstructure and its descendants like the replication and division hyperstructures may be determined by energy metabolites like ATP and GTP, alarmones like (p)ppGpp, and redox molecules like glutathione. Importantly, the presence of a hyperstructure may help us understand how the interactions between replication enzymes and metabolic enzymes occur and affect chromosome replication (Norris et al., 2007a; Soultanas and Janniere, 2023; Baranska et al., 2013). For example, the presence and conformation of the glycolytic enzyme, pyruvate kinase, in the initiation hyperstructure (at least, in *B. subtilis*) might allow metabolic information to be transduced by this hyperstructure into the timing of initiation and, subsequently, by the replication hyperstructure into the speed of elongation. In this context, it is worth noting that the belief in a constant speed of elongation is based on analyses of populations; given that single cell analysis has revealed the effect of gene dosage on transcript levels (Pountain et al., 2024), analysis of individual cells based on *DNA combing* and *Secondary Ion Mass Spectrometry* (Cabin-Flaman et al., 2011) might reveal differences in replication speed in different regions of the chromosome that could influence the phenotype and generate meaningful diversity (Norris et al., 2017).

### 3.6 Hypotheses on oscillation

#### 3.6.1 ATP and other small molecules

What might be the physical nature of the oscillation that causes the Initiation of replication? One possibility is an oscillation in the level of ATP, leading perhaps to an oscillation in the level of GTP and other molecules. In mammalian cells, metabolic oscillations have been proposed as fundamental to the regulation of the cell cycle (Papagiannakis et al., 2017); in yeast, such oscillations were not observed (Takaine et al., 2019) though it was found, significantly, that low levels of ATP could cause protein aggregation (Takaine et al., 2022). In *E. coli*, cells can have large fluctuations in the ATP level that are partially coordinated with the cell cycle (Lin and Jacobs-Wagner, 2022) and a corresponding activation of DnaA combined with its titration have been proposed as responsible for timing initiation at all growth rates (Berger and Wolde, 2022). A related possibility is a redox oscillation, which occurs in the cell cycle of human and yeast cells with the entry into and progression through the S phase occurring during the most reductive phase of this cycle [Klevecz et al., 2004; for other references see (Soultanas and Janniere, 2023)]; this metabolic oscillation may also cause general changes including in the central carbon pathways (Soultanas and Janniere, 2023). Similarly, in *Caulobacter crescentus*, fluctuations in metabolites such as glutathione occur during the cell cycle (Hartl et al., 2020) that are necessary for the optimal functioning of ribonucleotide reductase during elongation and for the inactivation of reactive oxygen species generated during the oxidative phase.

#### 3.6.2 A water clock?

**Figure 3:**
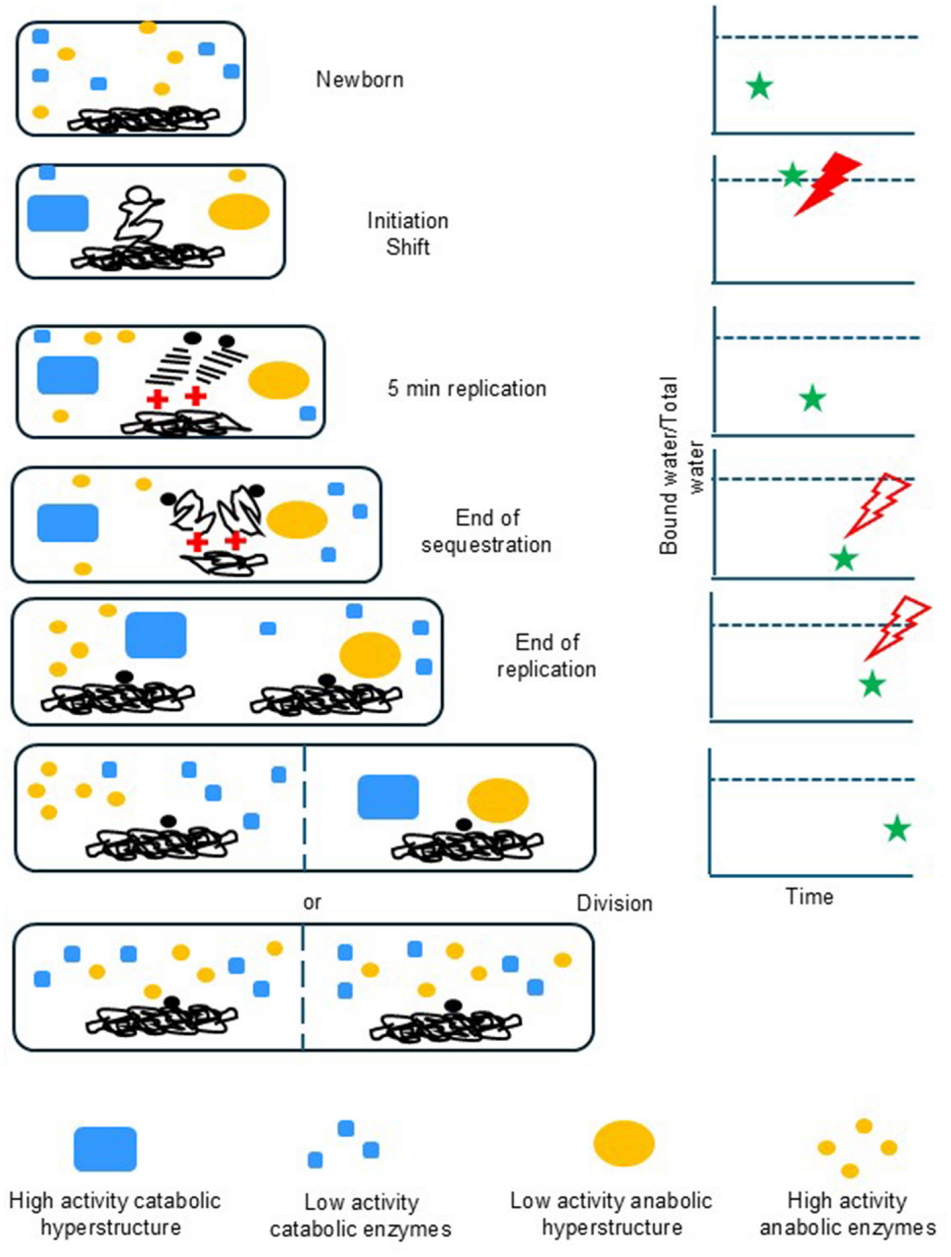
A possible phase transition scenario for the cell cycle. In this scenario, which is one of several that are complementary (Norris, 2024b), a subset of catabolic enzymes (blue rectangles) has an activity that increases when they are in a hyperstructure whereas this situation is reversed for a subset of anabolic enzymes (yellow ellipses), which has an activity that decreases when they are in a hyperstructure. Hyperstructure assembly occurs when the ratio of bound water to total water (green star) exceeds the phase transition threshold (dotted line in right panels); when a hyperstructure is assembled, water is released and the ratio drops. Below the threshold, anabolic activity dominates so as to make an increasing number of enzymes that bind an increasing number of water, molecules (which increases the water ratio). Above the threshold, catabolic activity dominates so as to make an increasing number of free water molecules. Other hyperstructures can also form above the phase transition threshold; these include those responsible for the replication of the chromosome (black lines), with either one or both nascent daughter chromosomes adopting a condensed structure that releases water (the origin region is the filled black circle, the replisomes are red crosses). Once created, hyperstructures persist for a significant part of the cell cycle. Oscillations in small molecules may result from the changes occurring in water availability at the initiation phase transition (red jagged shape) or possibly at other times (empty red jagged shapes). Phenotypic diversity could be generated by the unequal segregation of hyperstructures.

Another possibility for the physical nature of the oscillation is the water potential (essentially, the availability of water) itself (Norris, 2024b). In the formation of condensates, IDPs are particularly responsive to the structure of water (Zaslavsky and Uversky, 2018). A decrease in the availability of water during growth might therefore be transduced by IDPs. To give a simple example, suppose the water-binding products of anabolism accumulate during growth. The resulting change in water availability might cause a phase transition—the *Initiation Shift*—and the assembly of an initiation hyperstructure (along with other condensate-based hyperstructures); the assembling into condensates would release water and restart the cell cycle. This “water-clock” hypothesis has the advantage of explaining how catabolism and anabolism could be balanced and how the opening of DNA strands could be coupled to growth (Figure 3). The release of water aspect of the hypothesis is supported by the finding that water is released by the formation of a condensate (Watson et al., 2023). That said, a problem for the water-clock hypothesis is that its proposed regulation of the cell cycle by water availability has to take account of the fact that either a drop in temperature or an increase in osmolarity can cause a condensate to form and rapidly release water (Watson et al., 2023). This is reminiscent of a fundamental problem in chemotaxis whereby a cell is able to swim up a gradient of an attractant despite these gradients differing by up to five orders of magnitude; encouragingly, Bray found an elegant solution to this problem based on the dynamics of hyperstructures whereby, at high concentrations of an attractant, the receptors function independently, whilst, at low concentrations, the receptors cluster to pass information between them and amplify the signal (Bray et al., 1998).

### 3.7 Testing the concept

How can we prove that an initiation hyperstructure actually exists? One way to identify its constituents would be to examine the effects of hyperosmotic shock, which provokes plasmolysis with membrane distortion in different types of mutants. Meury et al. (1995) found that administering a hyperosmotic shock to *dam* cells led to an arrest of cell division accompanied by filamentation. Since the same type of filament is produced in a *seqA* mutant as well as in an *aph*A mutant (which is defective in another hemimethylated DNA binding protein; Reshetnyak et al., 1999), a deficiency in the sequestration of *oriC* in the membrane may be responsible for the inhibition of cell division (Bahloul et al., 1996). This osmotic shock approach may prove useful in finding other gene products that participate in the initiation hyperstructure.

Another classical way to analyze a multi-protein complex is to perform microscopy on cells producing fluorescence-tagged proteins. This might reveal a sequence in which the different constituents are added to the hyperstructure. In particular, if a constituent joins the hyperstructure as a condensate, fluorescence microscopy might detect it as a focus and, in the case of an initiation hyperstructure, might be expected to have its intensity vary during the cell cycle.

Other techniques that might be used to analyze hyperstructures include *Secondary Ion Mass Spectrometry*, which can reveal the close proximity of different macromolecules (to within a couple of nm) if one is labeled with ^13^C and the other with ^15^N (Legent et al., 2008), and *toponomics*, which, astonishingly, can localize up to a 100 different types of proteins to within a few tens of nm (Schubert et al., 2012).

The main objective of the above experiments would be to test the hypothesis that the initiation-sequestration hyperstructure interacts with *oriC* in order to enable Fts-Z to prime the formation of a constriction ring at the membrane site to which *oriC* is connected directly or indirectly. Based on this hypothesis, it may prove possible to isolate a mutant of *oriC* that has problems in the normal cell division process. In order to analyze *oriC*-DnaA interactions, Grimwade et al. (2007) have constructed several types of *oriC* mutants that may prove relevant whilst, encouragingly, Vinella et al. (1992) observed anucleate cell production in an *oriC* sequestration-deficient, *dam* mutant.

## 4 Discussion

The last 60 or so years have seen an enormous advance in our understanding of the initiation of chromosome replication in bacteria like *E. coli*, largely due to experiments and hypotheses based on molecular genetics and biochemistry. Good examples of these hypotheses include the Replicon Theory and the Initiation Mass. They have guided experiments that have generated results (see above) that are sometimes difficult to reconcile with these hypotheses and that may require the formulation of new hypotheses as licensed by the field (Norris et al., 1994) or, put differently, that require a paradigm shift (Kuhn, 1962; Cooper, 2017).

**Figure 4:**
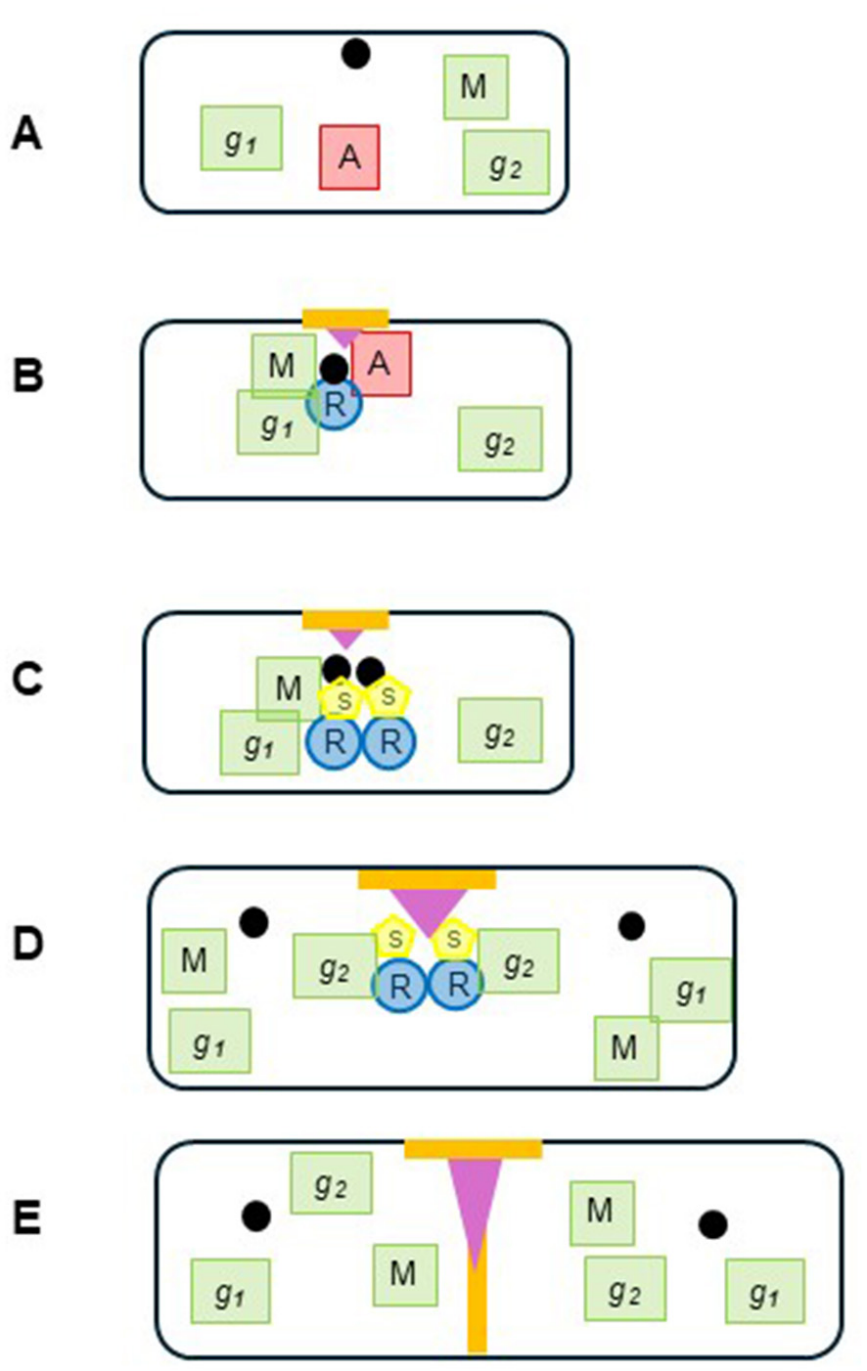
Trajectory of the replication hyperstructure. **(A)** Before initiation, some anabolic and catabolic activities (M for metabolic enzymes; *g1* and *g2* for gene expression) are in the cytoplasm. DnaA (A in red square) distributed away from the origin (black circle); **(B)** A phase transition causes an initiation hyperstructure to form in the center of the cell comprising a membrane domain (orange bar), the origin, DnaA, the replisome enzymes (R in blue circle), metabolic enzymes (like those providing precursors for DNA replication and those responsible for central carbon metabolism) along with the genes encoding them (*g1*), plus division enzymes like MinC and FtsZ (purple triangle); **(C)** During the early stages of replication, what is now the replication hyperstructure also contains SeqA (S in yellow circle). DnaA has left the hyperstructure; **(D)** At the end of the sequestration period, the origins, metabolic enzymes and the *g1* genes encoding them leave the hyperstructure whilst other *g2* genes join; **(E)** At the end of replication, the hyperstructure turns into a division hyperstructure with the arrival of new division enzymes.

A paradigm shift in the field of the bacterial cell cycle is well underway (Norris, 2019). It is taking the form of experiments and hypotheses that are now more closely based on physical chemistry and physics. The level of the key players in the cell cycle has been proposed to be intermediate between the macromolecule and the cell; it comprises large, extended structures of molecules and macromolecules termed hyperstructures that serve a function and that can undergo a developmental trajectory (Norris et al., 2007a). One of these hyperstructures is, we have proposed, the DnaA-based initiation hyperstructure, which may act as a logic gate integrating different metabolic signals [via, for example, the participation of enzymes of the central carbon metabolism (Soultanas and Janniere, 2023)] and pursue a trajectory that would include it maturing into a division hyperstructure via rich and complex changes in its structure and composition (Herrick et al., 2025; Figure 4). This kind of idea is supported by results from the explosion of work by physical chemists and their collaborators on condensates [for references see (Rivas and Minton, 2022)], which have led to phase transitions being invoked in the assembly and activity of FtsZ (Ramm et al., 2023) and, hypothetically, being involved in the formation of an initiation hyperstructure via condensation-prone IDPs (Herrick et al., 2025). However, the theoretical and experimental toolkit that physical chemists carry contains much more. It contains, for example, polyelectrolyte theory (the study of the behavior of charged polymers in solution), which in turn contains the theory of Oosawa-Manning ion condensation whereby counterions can diffuse in the “near region” along the polymer backbone (Manning, 1969; Oosawa, 1971); these counterions condense onto (and decondense from) linear polymers at a critical value of the charge density in what resembles a phase transition (Ripoll et al., 2004). This has led to the idea that ion decondensation from the *oriC* region might play a central role in initiation by favoring the opening of the strands (Norris, 2011; Norris and Amar, 2012). This idea is hence part of the physical chemical approach to the cell cycle in which cells live on the edge of phase transitions and in which initiation of replication is the result of the phase transition (Norris, 2024b) that we have termed here the *Initiation Shift*.

The paradigm shift occurring in the field of DNA replication may extend to cell division. The present paradigm for “non-differentiating” bacteria like *E. coli* is that a cell in steady state growth divides to give two daughters that are essentially identical. It has long been evident on theoretical grounds that this is improbable. Cells contain circuits that are subject to globally negative regulation and locally positive regulation: differentiation should therefore be considered inevitable unless mechanisms have evolved to prevent or reverse it (Norris and Madsen, 1995). One possibility is that the cell cycle organizes hyperstructures so as to yield two different coherent phenotypes, one (containing non-equilibrium hyperstructures) suitable for growth and the other (containing equilibrium hyperstructures) suitable for survival (Norris, 2015). To restore a maximal growth rate to a population of bacteria like *E. coli* in exponential growth, it might be expected that these phenotypes should be reversed if conditions remain unchanged. It is worth noting, however, that if a set of equilibrium hyperstructures follows one parental strand and a set of non-equilibrium hyperstructures follows the other parental strand, a considerable growth rate diversity can be generated epigenetically at little cost to the average growth rate of the population (Gangwe Nana et al., 2018), which may help explain why replication is semi-conservative (Norris and Ripoll, 2021). In line with the role of the asymmetric segregation of hyperstructures in the phenotype, it has been reported that, as *E. coli* leaves exponential growth, it starts to divide asymmetrically and only one of the daughters contains a glycogen condensate thereby, perhaps, equipping it for survival (Thappeta et al., 2024). A similar reasoning may be relevant to the role of polyphosphate, which, during the stringent response, activates the Lon protease that then degrades DnaA-ADP but not DnaA-ATP (Gross and Konieczny, 2020); it turns out that, *in vitro*, polyphosphate together with the nucleoid-associated protein, Hfq, interact with AT-rich DNA sequences to form phase-separated condensates as mediated by an intrinsically disordered region (Beaufay et al., 2021). Finally, the results of TRIP demonstrate the coordination of the expression of *E. coli* genes with their duplication (Pountain et al., 2024), which starts at *oriC*. It is not therefore surprising that displacement of *oriC* (von Meyenburg et al., 1979) or addition of another *oriC* (Wang et al., 2011) or reliance on an *oriK* (Ogawa et al., 1984) disturbs steady state growth: *oriC* is the keystone of the cell cycle.

## 5 Conclusion

**Figure 5:**
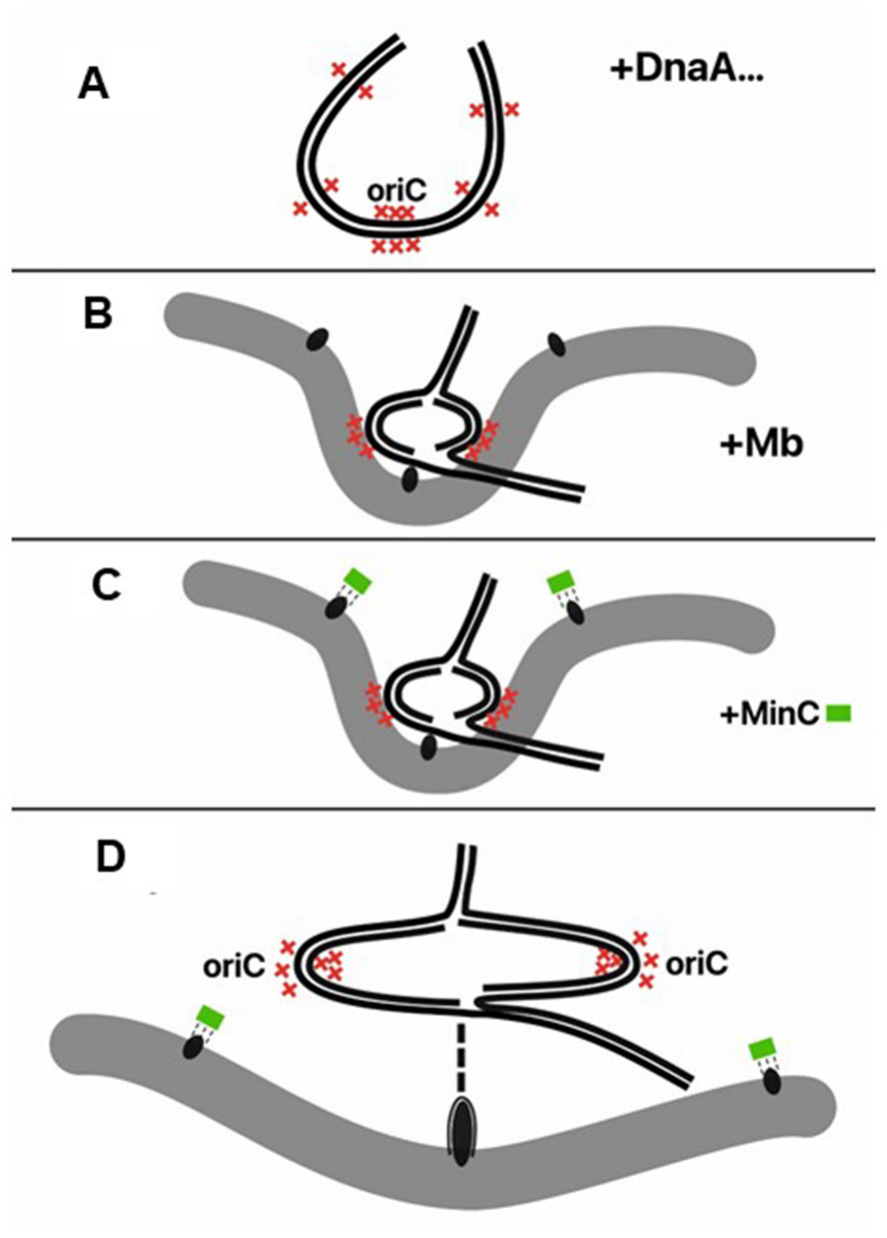
Membrane memory. **(A)** An initiation hyperstructure (containing DnaA, IHF, Fis, DnaB, DnaC etc.) opens *oriC*. Red cross represents Dam methylation site; **(B)** Hemimethylated *oriC* binds to membrane (gray band, membrane; black ellipses, FtsZ); **(C)** MinC (green box) represses FtsZ at membrane except the one protected by sequestrated *oriC* [like the MinC around the septum (Rothfield et al., 2005)]; **(D)** Two fully methylated *oriCs* released from membrane move quickly to opposite directions. FtsZ in the sequestration region develops the constriction ring with other future septum elements.

The Replicon Theory and the concept of an Initiation Mass (now initiation cell length) have, via *oriC*, played the leading role in guiding the thinking and experiments of generations of biochemists, geneticists and molecular biology. They have resulted in the discoveries of the multiple actions and mechanisms of *oriC* and its partner, DnaA, that implicate the DUE, DnaA boxes, sites for the binding of nucleoid-associated proteins like IHF and FIS, phospholipids, topoisomerases, DAM methylation and SeqA. We suggest that *oriC* will continue to play an important role but in the context of a hyperstructure that starts as an initiation hyperstructure based on *oriC* that then follows a developmental trajectory through a sequestration hyperstructure, again involving *oriC*, a replication hyperstructure, and finally a division hyperstructure. More precisely, we may imagine the following processes: the duplication of *oriC* within the initiation hyperstructure (containing DnaA and DNA duplication factors) and then *oriC* sequestration by the hyperstructure containing hemimethylated DNA binding proteins (SeqA and AphA) associated with membrane and with FtsZ. This *oriC*-associated FtsZ is perhaps indifferent to the inhibiting action of MinC [unlike those lying on the cell axis (Rothfield et al., 2005)] and continues to drive constriction after the end of sequestration (Figure 5). In this way, *oriC* marks the site on the membrane for FtsZ to start to form the division site.
